# Juvenile-onset Systemic Lupus Erythematosus: Recent Advances in Pathogenesis and Treatment

**DOI:** 10.1007/s11926-025-01207-7

**Published:** 2025-12-18

**Authors:** Valentina Natoli, Amandine Charras, Eve MD Smith, Christian M. Hedrich

**Affiliations:** 1 Department of Women’s and Children’s Health, Institute of Life Course and Medical Sciences, University of Liverpool, Liverpool, UK; 2 Dipartimento di Neuroscienze, Riabilitazione, Oftalmologia, Genetica e Scienze Materno-Infantili, Università degli Studi di Genova, Genoa, Italy; 3 Rheumatology and Autoinflammatory Diseases Unit, IRCCS Istituto Giannina Gaslini, Genoa, Italy; 4 School of Infection & Immunity, University of Glasgow, Glasgow, UK; 5 Department of Paediatric Rheumatology, Alder Hey Children’s NHS Foundation Trust Hospital, Liverpool, UK

**Keywords:** Juvenile-onset, SLE, Monogenic, Lupus, Genetic, Treatment

## Abstract

**Purpose of Review:**

This review summarizes recent advances in understanding the pathogenesis and therapeutic landscape of juvenile-onset systemic lupus erythematosus (jSLE), with a focus on how emerging genetic and immunologic insights inform patient stratification, targeted treatments, and Treat-to-Target (T2T) approaches in pediatric care.

**Recent Findings:**

Studies of (ultra-)rare gene variants (e.g., affecting *TLR7*,* UNC93B1*,* PLD4*,* PTPN2*,* BACH2*) aided in understanding key pathogenic pathways, and allowed linking these to associated clinical phenotypes. Multi-ancestral genomic studies and cumulative genetic metrics are refining links between patient ancestry, disease expression, genetic burden and variability, supporting personalized management. The therapeutic armamentarium has expanded with the approval of the first two biologic agents for SLE, belimumab and anifrolumab, alongside emerging molecular therapies such as protein kinase inhibitors (including JAK inhibitors), and new approaches to lupus nephritis induction using multitarget regimens that combine standard therapy with belimumab or calcineurin inhibitors. Early experience with CD19-directed CAR-T cells promises remarkable efficacy with sustained drug-free remission and good short-term safety in refractory SLE, although long-term outcomes remain under evaluation. Pediatric T2T strategies have been adapted to jSLE, and achievement of these targets are associate with improved disease control and reduced long-term damage.

**Summary:**

Recent findings confirm that converging genetic variants and immune pathway dysregulation underlie the heterogeneity of jSLE, supporting precision management. Advances in biologic and cellular therapies, together with paediatric T2T strategies, promise to improve outcomes. Future priorities include integrating genomic stratification into clinical practice and conducting dedicated pediatric trials of novel targeted therapies.

## Background

Systemic lupus erythematosus (SLE) is a chronic autoimmune/inflammatory disease with significant morbidity and mortality [1, 2]. Disease severity and outcomes vary between ancestries and age-groups. Patients of non-European ancestry and those with disease-onset during childhood/adolescence are most severely affected [3–5]. Indeed, 15–20% of patients with SLE develop disease before their 18th birthday. Patients with juvenile-onset (j)SLE, when compared to adult-onset disease, are more severely affected and exhibit higher disease activity and pre-existing organ damage at diagnosis, accrue more damage over time, require more aggressive treatment, and exhibit higher mortality rates [3, 4, 6].

While the exact molecular pathophysiology of SLE remains unknown, genetic factors have been identified as key contributors [1, 3–5, 7]. In most SLE patients, genetic variation increases disease risk and additional factors, such as hormonal changes and environmental exposure, are necessary for disease expression [1, 3–5]. This links to the increased incidence and prevalence after puberty and the female predominance typical for SLE (across pediatric patients 4:1, after puberty 9–10:1) [4, 5, 8]. The significant genetic contribution and genetic variability in the context of environmentally mediated factors likely explain clinical and phenotypic differences between patients, ancestries, geographic regions and age-groups [4, 7, 8]. Notably, only a small proportion of patients with SLE (1–3%) exhibit disease caused by single or the combination of few damaging gene variants with high impact on gene function [4, 8, 9]. These diseases have been summarized as monogenic SLE. While single gene variants do not explain SLE in the majority of patients, their study significantly improved our current understanding of SLE, especially in children and young people where genetic factors are ‘stronger’, contributing to early disease onset and severe phenotypes [3, 9, 10].

In the following, we will discuss recent reports on the molecular pathophysiology of jSLE and how they can improve diagnosis and care (without claiming to be fully exhaustive).

## Understanding Disease Mechanisms To Guide Patient Stratification

### The Contribution of Rare Variants

Across age groups, a small proportion of SLE patients (estimated 1–3%) experience disease caused by (ultra-)rare mutations in single genes with minor allele frequencies < 1% [5, 11, 12]. Notably, among children, especially in those with pre-pubertal disease onset, monogenic disease is more common and single centers and consortia reported rare SLE-associated variants in 3.5–15% of jSLE patients [10, 13, 14].

While rare variants are an important source of heritability, they are not covered by genome-wide association studies (GWAS) [12]. Some gene variants were first reported in association with other diseases with an inflammatory component (e.g., Aicardi-Goutières syndrome (AGS), stimulator of interferon genes (STING)-associated vasculopathy with onset in infancy (SAVI), Noonan syndrome, etc.) and linked with SLE later [8–11, 15] **(**Table 1 summarizes monogenic jSLE causes reported since 2020). In the following, we will touch on a non-exhaustive selection of recently reported ‘monogenic’ causes of SLE.

**Table 1:** ‘Monogenic’ SLE reported since 2020

Gene	Variant	Ancestry	Patient characteristics	Functional studies and/or immune phenotyping	Ref.	Pathway
ACP5	c.155 A >C/p.K52T (homozygous)	Turkish	Two siblings with SPENCD and jSLE features Onset at 2 and 19 years consanguineous parents.	None	[16]	ACP5 plays a role in shaping the immune balance between Th1 and Th2 responses, and regulatory T cell (Treg) function.
ADAR	c.2815 A >G, p.Ile939Val (heterozygous)	Croatian	15-year-old girl with jSLE. Clinical manifestations included neuropsychiatric lupus, LN, coagulopathy, immunodeficiency and features of malformation syndrome. Family history included malformation syndromes and unexplained early deaths.	PBMCs analyses did not reveal pathological type I IFN signature.	[17]	*ADAR* encodes for adenosine deaminases that edit double-stranded RNA by converting adenosine to inosine, thereby regulating RNA stability, splicing, and innate immune responses.
BACH2	c.1727G >T/p.R576L (heterozygous)	Chinese	Girl with SLE and LN (multiple autoimmune symptoms, recurrent infections, fungal infection, and poor treatment response) Onset at 4 years Variant inherited from parent	Decreased serum IgA, absence of IgA + memory B cells, deficient transcriptional repression of BLIMP1 and limited IgA class switch recombination (CSR) demonstrating that BACH2 inhibits B cell differentiation and promotes CSR by repressing BLIMP1. Moreover, activation of the BAFF/APRIL system, expansion of CD21^low^ B cells, and high expression of ICOS on CD4 and follicular helper T cells.	[18]	BACH2 is a key regulator of adaptive immunity, crucial for the maintenance of regulatory T cell function and B cell maturation.
BLK	c.211G >A/p.Ala71Thr (heterozygous)	Croatian	17-year-old girl with jSLE affecting the heart, kidneys, gastrointestinal system, pancreas, musculoskeletal system and serous membranes. The patient’s mother has SLE, and antiphospholipid syndrome; her sister has a child with juvenile localized scleroderma. Additional heterozygous variant in *C1R* (c.4052dupC).	None	[17]	BLK belongs to a Src kinase family involved in B cell receptor (BCR) signalling, B cell development, and immune regulation.
C8	rs373473282, p.Arg480His	Swedish	Patient 3, 14 years old, was positive for ANA, dsDNA, ACL and presented Infections, Libman-Sacks endocarditis, haemolytic anaemia, arthritis, SS. Patient had large number of organs damaged and died of pneumonia. Patient carried another mutation on *IFIH1* (rs376048533, p.Arg822Gln*)*	None		Complement pathway
CD3G	c.213delA/p.Lys71fs (homozygous)	Chinese	10-year-old boy with SLE-like disease, autoimmune thyroiditis, asthma, immunodeficiency, recurrent infections.	None	[19]	Part of the CD3/TCR complex that plays an essential role in adaptive immune responses.
COPA	c.2294 A >G/p.His765Arg (heterozygous)	Oman	3 siblings (girls), including - one patient with LN. - one clinically healthy girl who tested positive for ANA but remained negative for antibodies to ENA, dsDNA and APLS. - one sibling with no symptoms or autoantibodies.	None	[20]	*COPA* encodes for the coatomer subunit alpha that is involved in retrograde transport from the Golgi apparatus to the endoplasmic reticulum, maintenance of protein trafficking and ER homeostasis.
DDX58	rs147964586/p.Ala30Val	Swedish	Patient 5: 17-year-old patient with LN (WHO class IIIB), positive for ANA, dsDNA, ACL, B2GPI; developed malignancy later.	None	[21]	The *DDX58* gene encodes for RIG-I (retinoic acid–inducible gene I), a cytosolic sensor for viral double-stranded RNA that triggers IFN production and initiates innate antiviral immune responses.
	rs138425677/p.Pro885Ser	Swedish	Patient 6: 17-year-old patient with rash, arthritis, serositis, mildly reduced glomerular filtration rate, tested positive for ANA and dsDNA antibodies.	None	[21]	
DNASE1L3	c.290_291delCA/p.Thr97Ilefs*2 (Homozygous)	Turkish	15-month-old patients with urticarial rash, often precipitated by milk and wheat consumption, immune-complex glomerulonephritis with ANA, anti-dsDNA, and ANCA positivity. Patient had a deceased brother (at 17 years) who experienced similar symptoms.	None	[22]	Involved in the degradation of extracellular DNA from apoptotic or necrotic cells.
	c.290_291delCA p.T97Ifs*2 (Homozygous)	Italian	5-year-old boy with steroid-resistant nephrotic syndrome, membranous ‘full-house’ glomerulonephritis.	MXA expression (IFN surrogate) in glomeruli of kidney biopsies. IFN signature in blood samples taken during active disease; reduced DNAse activity.	[23]	DNASE1L3 is involved in the degradation of extracellular DNA, particularly DNA released from dying cells due to apoptosis or necrosis.
	c.290_291delCA p.T97Ifs*2 (Homozygous)	Italian	4-year-old boy with SLE, haemolytic anaemia, arthralgia, glomerulonephritis, nephrotic syndrome at 7 years.		[23]	
	c.290_291delCA/p.T97Ifs*2 + ex 5 del	Italian	4-year-old girl with chronic kidney disease.		[23]	
	c.289_290delAC/p.Thr97Ilefs*2 resulting in large deletion of exon 5	Albanese	Patient 1 is 4 years old girl. She had recurrent episodes of fever associated with fatigue, a polymorphic erythematous rash and non-erosive arthritis of the hands and feet. Laboratory tests revealed haemolytic anaemia, increased ESR and CRP, elevated LDH, and hypocomplementemia. She had antinuclear antibodies (ANA) and anti-dsDNA antibodies.	Patients showed variable induction of type I interferon signalling as assessed by nanostring and RT-qPCR (whole blood RNA extraction).	[24]	
	homozygous c.433G >A (both parents heterozygous carriers)	Arab	11-year-old girl with bilateral arthralgia, chronic atypical urticaria, lupus-like glomerulonephritis with increased serum creatinine, moderate proteinuria and haematuria. Autoantibody profile included ANA, ANCA with anti-MPO specificity.		[24]	
	c.289_290delAC/c.321-1G >A	Italian	15-year-old girl with recurrent fevers, urticaria, arthralgia, myalgia, and severe anaemia. Positive for ANA.		[24]	
	homozygous c.653delT	French (?, not clearly stated)	18-month-old boy with abdominal pain, vomitus and diarrhoea, recurrent urticaria accompanied by a macular rash, arthritis. He had leukocytoclastic vasculitis and chronic inflammation of the intestine with eosinophilic infiltration.		[24]	
	Homozygous c.581G >A (p.Cys194Tyr)	French (?, not clearly stated)	4-year-old girl with lower limb pain, severe anaemia, proteinuria, haematuria, hypertension, and acute kidney injury as a result of immune complex glomerulonephritis and small-vessel vasculitis. Laboratory tests revealed hypocomplementemia, anti-C1q antibodies, and ANCA positivity.		[24]	
DNASE1L3	c.643delT/p.Trp215GlyfsX2 (homozygous)	Arabic from Oman	15 families: The monogenic lupus cohort showed a young age at onset (mostly before 5 years). Patients presented with arthritis (82%), nephritis (60%), urticarial rash (79%), conjunctivitis (36%), abdominal pain (36%), and pulmonary haemorrhage (15%). In most cases (88%), the urticarial rash appeared months before other SLE features (median 9 months). The cohort showed high rates of anti-dsDNA positivity, low complement levels, and ANCA positivity (28%).	None	[25]	The DNASE1L3 is involved in the degradation of extracellular DNA, particularly DNA released from dying cells due to apoptosis or necrosis.
DNASE2	c.1040G >A/p.Cys347Tyr (homozygous)	Indian	6-year-old boy with global developmental delay, microcephaly, chronic fevers, anaemia, rash, polyarthritis, renal involvement, hepatosplenomegaly.	None	[26]	Plays a role in the clearance of nucleic acids generated through apoptosis.
IFIH1	rs775244788, p.Val361Ile	Swedish	Patient 1, 3 years old, was positive for ANA, dsDNA, SSA, ACL, LA and presented Stroke, Seizures, lung capillaritis, APS and developed cognitive impairment after study.	None	[21]	IFIH1 is cytosolic sensor of viral double-stranded RNA that activates type I interferon responses and innate antiviral immunity.
	rs376048533/p.Arg822Gln	Swedish	Patient 3: 14-year-old patient with recurrent infections, Libman-Sacks endocarditis, haemolytic anaemia, arthritis, multiple organ damage, died of pneumonia. Patient carried another mutation in *C8A* (*rs373473282/p.Arg480His)*	None	[21]	
IKZF1	rs117111762/p.His119Arg	Swedish	Patient 7: 17-year-old patient with arthritis, seizures, headaches.	None		*IKZF1* encodes for IKAROS, a zinc finger transcription factor that plays a key role in lymphocyte development and immune regulation.
IKZF2	c.1536T >G/p.Tyr512Ter (heterozygous)	Arabic from Oman	2 siblings, one had jSLE (boy), the other had primary anti-phospholipid syndrome (girl). 5 remaining siblings are carriers with no symptoms.	None	[20]	The *IKZF2* encodes for Helios, a zinc finger transcription factor of the Ikaros family that plays a key role in T cell differentiation, particularly in the development and function of regulatory T cells.
NLRC4	c.1964G >C, p. W655S (heterozygous)	Chinese	A 19-year-old Chinese woman (patient 1) with recurrent fevers, diarrhoea and moderate to severe autoimmune haemolytic anaemia, splenomegaly, lymphadenopathy, and pleural effusion. Her son (patient 2), developed neonatal lupus syndrome with macrophage activation; he died at 27 days of age.	Increased IL-1 and IL-18 in the serum and following PBMCs stimulation with LPS and LPS + FLA-ST (NLRC4 inflammasome activator). RNA-sequencing analyses of patient PBMCs indicated enhanced inflammatory signalling.	[27]	*NLRC4* encodes NLR family CARD domain–containing protein 4, an intracellular pattern recognition receptor that forms the NLRC4 inflammasome, activating caspase-1 and promoting the release of IL-1β and IL-18 in response to bacterial infection and cellular stress.
PLD4	c.C542T/p.P181L and c.C968T/p.A323V	Chinese	Patient 1: 12-year-old patient with periorbital oedema following tonsillitis, decreased urine output, proteinuria, haematuria and hypoalbuminemia. Two months later, diagnosed with jSLE, diffuse proliferative LN.	The five variants reported (three in children and young adults) associate with functional impairment of PDL4, affecting the protein’s exonuclease activity. This impacted the cGAS-STING signalling with resulting IFN expression. A mouse model confirmed the central role of PLD4 in the development of SLE. Authors corrected clinical phenotypes observed in Pld4⁻/⁻ mice using the JAK inhibitor baricitinib.	[28]	PLD4 encodes for a 5’>3’ exonuclease involved in the degradation of single-stranded DNA and RNA.
	c.C567A/p.D189E and c.G1370A/p.G457D	Chinese	Patient 2: 16-year-old girl with SLE and LN. History of urticaria, alopecia, proteinuria, haematuria, elevated serum creatinine, leukopenia, thrombocytopenia, autoimmune haemolytic anaemia, pericardial and pleural effusions, and lymphadenopathy.		[28]	
	c.G602A/p.R201Q and c.1088delG/p.A364Rfs*45	Chinese	Patient 3: 19-year-old male with SLE, LN, arthritis.		[28]	
PTPN2	c.1209delT/p.Phe403Leufs*25 (heterozygous)	French	5-year-old girl with SLE and skin rash, lupus nephritis, hematological involvement, chronic hepatitis and cholangitis (Metavir score A3F3). Positive antinuclear antibodies (ANA), anti-β2 glycoprotein-1 (β2GP1),and anti-C1q.	All six variants reported in this manuscript led to functional deficiency of PTPN2, either by loss of expression or by changes in its phosphatase activity resulting in hyperactivation of the JAK/STAT pathway.	[29]	Negative regulator of the JAK/STAT pathways
SAT1	c.118G >T/p.Asp40Tyr (hemizygous, inherited from mother)	American African	6-year-old boy with jSLE, malar rash, nonerosive arthritis, serositis, pleuritis, pericarditis and LN.	The authors demonstrated that Tyr40-containing constructs produced two aberrantly spliced transcripts: one with exon 2 skipping, leading to premature termination of translation, and another with intron 2 retention, which also impaired protein function. For the other identified variant, the authors generated Sat1p.Glu92Leufs*6 knock-in (KI) mice (C57BL/6J background) using CRISPR/Cas9 technology. Mice spontaneously developed lupus-like features, including splenomegaly, elevated levels of IgG anti–double-stranded DNA antibodies, proteinuria and LN. Functional studies in mice, revealed that bone marrow–derived neutrophils displayed spontaneous NETosis and a defect in autophagy. And mice presented alterations in Foxp3-related T cell subsets.	[30]	SAT1 catalyses the acetylation of polyamines and is involved in their transport out of cells.
	c.118G >T/p.Asp40Tyr (hemizygous, inherited from mother)	American African	7-year-old boy with jSLE, malar rash, leukopenia, lymphopenia and LN.		[30]	
	c.272_273dup/p.Glu92Leufs*6 (hemizygous, inherited from mother)	American African	12-year-old boy with jSLE, nonerosive arthritis, anemia, fatigue and LN		[30]	
	c.272_273dup/p.Glu92Leufs*6 (hemizygous, inherited from mother)	American African	8-year-old boy with jSLE, CNS lupus, nonerosive arthritis, retinal vasculitis and LN.		[30]	
SHOC2	c.4 A >G/p.Ser2Gly	Italian	13-year-old boy with Noonan-like syndrome and SLE with lymphopenia, thrombocytopenia, lupus nephritis, arthritis, serositis and immunological criteria (ANA, anti-dsDNA, antiphospholipid antibodies, lupus anticoagulant, low complement).	None	[31]	SHOC2 plays a critical role in the RAS/ERK MAP kinase signalling, acting as a scaffold that links RAS to downstream effectors including RAF proteins.
	c.4 A >G/p.(Ser2Gly)/rs267607048	Turkish	2-year-old girl with SLE and antiphospholipid syndrome, features suggestive of Noonan-like syndrome.	None	[32]	
TLR7	c.800 C >T/p.P267L (heterozygous)	British	2-year-old girl with anti-NMDA encephalitis, large pericardial effusion, hemolytic anemia and SLE, vasculitis and seizures.	Laboratory work on the patient PBMCs indicated increased IFN-dependent proteins.	[33]	TLR7 recognizes single-stranded RNA in endolysosomes to induce IFN expression.
	p.Tyr264His; additional heterozygous variant in RNASEH2B (p.Ala177Thr)	Spanish	7-year-old girl with refractory autoimmune thrombocytopenia, ANA positivity and hypo-complememia who developed arthralgia, constitutional symptoms, intermittent episodes of hemichia, mild mitral insufficiency and renal impairment/hypertensive crisis.	In vitro and in vivo works notably on the mouse model developed indicate that this GOF mutation in TLR7 can cause B-cell-induced autoimmunity, including SLE in mice, which is thought to result from a lowering of the TLR7 activation threshold with this mutation. This would promote the survival of immature B cells activated by BCR and the activation of autoreactive B cells, without the need for T cell assistance	[34]	
UNC93B1	c.275 A >G/p.E92G (homozygous)	Madeiran	Two siblings with SLE from a consanguineous family: boy diagnosed at 4 months and a girl diagnosed at 2 years of age. Both had autoimmune thrombocytopenia, autoimmune anaemia and erythematous rash, followed by hepatosplenomegaly, glomerulonephritis, arthritis, multiple autoantibodies including ANA and dsDNA.	IFN signature and elevated pro-inflammatory serum cytokines from PBMCs. The cell type-specific transcriptional signatures and B cell phenotypes of patients carrying UNC93B1 mutations are very similar to those observed in genetically complex SLE. Type I IFN activation in patients carrying the UNC93B1 p.E92G or p.R336L mutation is likely caused by hyperactivation of TLR7. Unc93b1 p.E92G confers gain-of-function, selectively leading to hyperactivation of TLR7/8, but not TLR3 or TLR9. UNC93B1 p.R336L may interfere with the inhibitory function of UNC93B1 on TLR7, preventing the termination of TLR7 signalling, thereby leading to TLR7 hyperactivation.	[35]	UNC93B1 regulates trafficking of endolysosomal TLRs, their integration into the lysosomal membrane, function and degradation.
	c.1007G >T/p.R336L (heterozygous)	German	18-month-old boy with SLE (dad was diagnosed at 5 years), dermatitis, positive ANAs, hypocomplementemia, hepatomegaly, progressive generalized lymphadenopathy, and arthralgia, proteinuria, hyperglobulinemia, anti–β2-glycoprotein, and anti-dsDNA antibodies. His father, presented first symptoms at 5 years of age, including malar rash, photosensitivity, arthralgia.		[35]	
	c.1574_1575delinsCT/p. Arg525Pro (Heterozygous)	North African	Two boys & two girls across three generations with chilblain lupus.	Increased TLR7 activity with the p.I317M (in vitro) and p.G325C (in vitro and ex vivo); association with SLE. In contrast, in the three families with chilblain lupus, p.L330R, p.R466S and p.R525P conferred gain of TLR8 activity, with isomorphic TLR7 activity.	[36]	
	c.989T >G/p.Leu330Arg (Heterozygous)	European	Boy with chilblain lupus.		[36]	
	c.951 C >G/p.Ile317Met (Homozygous)	Indian	Girl with SLE, autoimmune haemolytic anaemia, Positive ANA, anti-dsDNA Aab		[36]	
	c.1398 A >C/p.Arg466Ser (Heterozygous)	European	Female patient with chilblain lupus.		[36]	
	c.973G >T/p.Gly325Cys (Heterozygous)	European	Female patient with SLE, pulmonary artery hypertension, cutaneous lesions on cheeks, autoimmune haemolytic anaemia		[36]	
	p.Glu49dup (Heterozygous)	-	7-year-old girl with progressive autoimmune symptoms, including atopic dermatitis, Hashimoto thyroiditis, sialadenitis, generalized lymphadenopathy, and splenomegaly, ANA, leukopenia, hypergammaglobulinemia, complement consumption.	An E49 duplication in UNC93B1 appears to be responsible for weakening the interaction between TLR7 and the BORC-ARL8B complex, which would lead to a reduction in degradative sorting and an accumulation of TLR7 in late endosomes. The patient’s allele exists as a monoallelic variant, which would indicate a dominant effect on TLR7 activity.	[37]	
	c.349G >T/p.V117L	Chinese	Multiple patients with SLE and LN.	Functional studies indicate that the identified UNC93B1 variants may intrinsically promote inflammation (THP1-monocyte model) and immune dysfunction (immortalised B cell lines) associated with type I IFN and NF-κB signalling pathways, via a TLR7/−8–IRAK1/−4 pathway. CRISPR-Cas9-carrier UNC93B1V117L mouse models developed lupus-like disease with organ damage and autoantibody production, comparable to the clinical picture of patients with the orthologous variant	[38]	

Only pediatric patients are described in this table jSLE: juvenile Systemic Lupus Erythematosus; Th1/Th2: T helper cells 1/2; Treg: T regulatory cells; LN: Lupus Nephritis; PBMCs: Peripheral blood mononuclear cells; RNA: Ribonucleic acid; CSR: class switch recombination; BCR: B cell receptor; ANA: Antinuclear antibodies; dsDNA: Anti-dsDNA antibodies; ACL: Anticardiolipin antibodies; TCR: T cell Recceptor; ENA: Extractable Nuclear Antigens; APLS: Antiphospholipid; ER: Endoplasmic Reticulum; B2GPI: Beta-2 Glycoprotein 1 Antibodies, RIG-I: retinoic acid–inducible gene I; ESR: Erythrocyte Sedimentation Rate; CRP: C-reactive protein; LDH: Lactate dehydrogenase; RT-qPCR: Reverse Transcription quantitative polymerase chain reaction; ANCA: Anti-neutrophil cytoplasm antibodies; MPO: myeloperoxidase; DNA: Deoxyribo Nucleic Acid; anti-SSA/Ro: anti–Sjögren’s-syndrome-related antigen A autoantibodies; anti-SSB/LA: anti–Sjögren’s-syndrome-related antigen B autoantibodies; IL-1/18: Interleukin 1/18; LPS: Lipopolysaccharides; FLA-ST: NLRC4 inflammasome activator; KI: knock-in; CNS: Central nervous system manifestations; anti-NMDA: anti- N-methyl-D-aspartate; GOF: Gain of Function

#### RASopathies and monogenic SLE

RASopathies are a clinically defined group of diseases caused by gene variants involving the RAS/mitogen-activated protein kinase (MAPK) pathway, including *SHOC2* (a positive regulator of mitogen-activated protein kinases (MAPKs). Extracellular signal-Regulated Kinase (ERK)1/2), *KRAS* and *NRAS* (GTP-ases), *PTPN11* (Protein tyrosine phosphatase nonreceptor type 11), *RAF* (Rapidly Accelerated Fibrosarcoma), and *SOS1* (Son of Sevenless 1) [39]. Resulting clinical phenotypes include Noonan, LEOPARD (Noonan syndrome with multiple lentigines), Costello and cardio-facio-cutaneous syndrome, neurofibromatosis type 1 (NF1) and Legius syndrome that share short stature, macrocephaly, subtly but distinct facial appearance, mild neurodevelopmental delay, cardiac and cutaneous manifestations [40]. Some are associated with an increased chance of malignancy or autoimmunity, including SLE [39].

Recently, *SHOC2* p.Ser2Gly (c.4 A >G; rs267607048) was reported in a 13-year-old boy with Noonan syndrome who later developed jSLE [31]. The same variant occurred in a 2-year-old girl with early-onset jSLE, antiphospholipid syndrome, and clinical features suggestive of Noonan syndrome [32]. This is of special interest as, also in more common genetically complex SLE, RAS/MAPK signaling is disrupted resulting in reduced ERK activation in T lymphocytes, DNA hypomethylation and increased CD40L expression which, in turn, promotes B cell activation, (auto-)antibody production and tissue inflammation/damage [41, 42]. Moreover, variants in *RASGRP1* (Ras guanyl-releasing protein 1), a RAS activator, increase susceptibility to SLE [43], and excessive activation of the RAS/MAPK pathway has been observed in renal tissue from patients with LN [44].

#### Intracellular Toll-like receptor (TLR) dysregulation in SLE

Toll-like receptor (TLR)7 dysregulation through defective X chromosome inactivation and resulting bi-allelic gene expression in women [45], *TLR7* duplication [46], common gene variants affecting gene expression [47–50], IFN gene: gene epistasis involving *TLR7* polymorphisms [51], and/or *TLR7* variants affecting transcript stability [52] has been linked with SLE. Recently, ultra-rare variants affecting *TLR7* [34, 53] or *UNC93B1* (Unc-93 homologue B1) [35, 37] have been linked with TLR7 gain-of-function in SLE.

Variation in *UNC93B1* has significantly improved our understanding of TLR processing and regulation of its activity. The UNC93B1 protein is a chaperone molecule for endolysosomal TLRs (TLR3,−7,−8,−9), facilitating their trafficking between the endoplasmic reticulum and the endolysosome [54]. It mediates their stable integration into the endolysosomal membrane which controls its activity [35], and is involved in TLR inactivation and degradation [37, 55]. Altered UNC93B1 function affects TLR7 sub-cellular trafficking, its integration into the endolysosome, and regulation of TLR7 activity and/or degradation, resulting in IFN expression and systemic inflammation [35, 37, 38, 55]. Notably, depending on the functional domain affected by damaging *UNC93B1* variants, defects impact more on TLR7 or TLR8 regulation which associates with differential clinical phenotypes. While *UNC93B1* p.Ile317Met (c.325G >C) associates with gain of TLR7 activity and jSLE, *UNC93B1* p.Leu330Arg, p.Arg466Ser, and p.Arg525Pro primarily associate with gain of TLR8 activity, isomorphic TLR7 activity and familial chilblain lupus [36]. This suggests that molecular stratification of patients with *UNC93B1* variants may inform future targeted therapeutic interventions (e.g., TLR7 versus TLR8 inhibition).

#### Dysregulation of type I IFN and cytokine signaling 

Defects in nucleic acid sensing and processing were among the first mechanisms reported to associate with pathologically increased type I IFN expression, systemic inflammation and autoimmunity [3, 10, 15, 56]. For example, loss-of-function variants affecting the 3’−5’ exonuclease *TREX1* (*DNAse3*) associate with familial chilblain lupus, early-onset SLE, and in AGS [57–59].

Recently, clinical phenotypes resembling (j)SLE have been linked with damaging variants in genes encoding for deoxyribonuclease (DNAse) enzymes that play an important role in innate antiviral immunity [15]. Deficiency of the cytoplasmic endonuclease of DNAse 1 (*DNASE1*) [60] or the lysosomal DNAse 2 (*DNASE2*) were reported in early-onset jSLE patients with somewhat ‘untypical’ features including neonatal pancytopenia, polyarthritis and cholestasis [26, 61]. The extracellular (serum) DNAse1L3 degrades antigenic cell-free DNA [62]. Mutations in *DNASE1L3* associate with SLE-like disease with nephritis and hypocomplementemic urticaria. Notably, in this disease, flares can be triggered by ‘stress’ inducing cell damage, such as infections, while type I IFN expression normalizes in phases of remission [24].

Type I IFNs are at the interface between innate and adaptive immune responses both of which are pathologically engaged in SLE. In a recent report, six previously not known monoallelic variants were discovered in *PTPN2* (protein tyrosine phosphatase non-receptor type 2), a critical negative regulator of Janus kinase (JAK)/signal transducer and activator of transcription (STAT) pathways involved in the regulation of type I IFNs and other pro-inflammatory cytokines [29, 63]. One patient of the cohort, carrying the *PTPN2* c.1209delT (p.Phe403Leufs*25) variant, experienced jSLE. All six variants studied impair PTPN2, either through loss of its expression or reduced phosphatase activity, subsequently resulting in enhanced JAK/STAT signaling [29].

The BACH2 (BTB Domain and CNC Homolog 2) transcription factor is involved in the regulation of B and T cell differentiation, function, and homeostasis including type I IFN signaling [64]. Recently, *BACH2* c.1727G >T (p.Arg576Leu) was reported in a patient with early-onset jSLE. In this patient, disease was characterized by significant immune dysregulation including low serum immunoglobulin (Ig)A, the almost complete absence of IgA^+^ memory B cells, deficient transcriptional repression of BLIMP1 (B lymphocyte-induced maturation protein-1, a transcriptional repressor involved in immune cell differentiation), and limited IgA class switch recombination. This suggests that BACH2 inhibits B cell differentiation while promoting class switch through the repression of BLIMP1. Moreover, activation of the BAFF/APRIL system, expansion of CD21^low^ B cells, and increased expression of the inducible T cell costimulator (ICOS) on CD4^+^ and follicular T helper cells underscore pathologically over-activated immune responses associated with *BACH2* c.1727G >T [18].

In summation, recent findings underscore the central involvement of RAS/MAPK, TLR, IFN, JAK/STAT and BACH2-associated pathways in the pathophysiology of SLE, underscoring the importance of studying monogenic disease for understanding immune biology and inflammatory disease.

### Common Gene Variants

In most patients, SLE is a genetically complex disease caused by the molecular interplay of common variants in the presence of hormones and environmental impact [4, 5, 7, 8]. Notably, SLE-associated common variants frequently affect genes that harbour deleterious mutations in monogenic SLE [1, 5, 8, 9]. Through the cumulative impact of common variants, the presence of (additional) rare variants with high(er) penetrance, hormonal changes (during puberty in girls) and/or the accrual of environmental exposure, inflammation and self-reactivity breach a threshold resulting in clinical disease [2, 56, 65].

GWAS have significantly improved our understanding of SLE. While previous studies have frequently been limited by their restriction to European ancestries [15], recent *meta-*GWAS stratified participants by ancestry [66–69] and/or performed *trans-*ancestral analyses [69–73] demonstrating shared and distinct SLE risk loci across populations [15]. Laurynenka et al. reported 330 risk loci for SLE, confirming the central role of genes associated with innate and adaptive immune responses, including the regulation of cytokines, type I IFN pathway, apoptosis, the detection/clearance of immune complexes, and immune responses to infections [74]. Notably, while some loci are specific to certain ancestral groups and genetic risk for SLE is highest among black ancestries, under-representation non-European participants is a common feature across the literature [15, 74]. In adult patients with SLE, common variants (minor allele frequency/MAF >5%) explain approximately 30% of the phenotypic variance in European populations and about 24% in East Asians [75]. In jSLE, where disease is more severe, both rare (MAF < 1%) deleterious gene variants and ‘common’ risk alleles are more common when compared to adult-onset (a)SLE, which may explain phenotypic differences [15, 76, 77].

The highly complex human leukocyte antigen (HLA) locus (chromosome 6) is the most polymorphic region of the genome [78]. It has strong associations with several inflammatory diseases and was the first genomic region associated with SLE [15, 79, 80]. A recent study across 11 autoimmune/inflammatory diseases (UK biobank) identified more HLA risk alleles in SLE when compared to other diseases [81]. The authors of this review recently confirmed genetic variation in the HLA cluster of jSLE patients that associates with organ involvement in an ancestry-specific manner. Risk associated with jSLE was conserved at related alleles HLA-DR2h (DRB1∗15:01, DQA∗01:02, DQB1∗06:02) and HLA-DR3h (C∗07:02 [Asian], B∗08:01, *C2* rs9332730 [Asian], DRB1∗03:01). Notably, HLA-DR7 haplotypes (DRB1∗07:01, OR = 0.44, 95% CI:0.27–0.72; DQA1∗02:01, OR = 0.34, 95% CI:0.21–0.56) protect Asians from jSLE development, and low complement C4 serum levels associate with HLA-DQA1∗01:02 (DR2h) in Europeans. An association between low white blood cell counts and HLA-A∗03:01P was present across ancestries. Observations confirm the complexity of HLA haplotypes in SLE that vary between ancestral groups, underscoring the importance of multi-ancestral approaches to genetic studies [82].

The central aim of studies investigating genetic variation in SLE is to associate genetic variability with an individual’s risk to develop SLE, clinical phenotypes, treatment response, complications, and/or disease outcomes [3, 8, 15]. Several studies have identified associations between genetic variation and LN, including a recent study involving pediatric and adult patients with SLE demonstrating associations between 39 non-HLA variants (OR = 1.26; 95% CI:1.09–1.46) and LN that were strongest among children of European ancestry [83]. Another study from Taiwan reported associations between a combination of 27 variants and LN at a young age [84]. Genetic associations with organ manifestations other than LN have been reported. We identified 10 type I IFN-related jSLE quantitative trait loci that associate with young age at disease-onset (*IRAK1*, *TLR7*, *IFIH1*, *STAT4*, *TYK2*, *IRF8*), global disease activity (*IFIH1*, *STAT4*, *IRF8*), and/or mucocutaneous involvement (*TLR7*, *IFIH1*) among jSLE patients from the UK [51]. A recent GWAS in a multi-ancestral cohort of juvenile-onset and adult SLE patients (*N* = 940 total; *N* = 71 osteonecrosis) established links between an intronic variant in *WIPF1* (WAS/WASL interacting protein family member 1) and increased osteonecrosis risk independent of glucocorticoid exposure [85]. Lastly, common variants in *TLR7* have recently been reported in jSLE patients from the UK, including three variants with impact on gene function: rs2302267/n.−20T >G (promoter); rs179008/p.Gln11Leu (loss-of-function); and rs3853839/c.∗881 C >G (3’UTR) [50]. *TLR7* variants associate with an individual risk to develop jSLE among girls of African/Caribbean ancestry (rs3853839_GC/GG; OR:1.8; 95%CI:1.2–2.9), while the same variant reduces risk in European girls (OR:0.5; 95% CI:0.4–0.7) [50]. Simultaneously, rs3853839 (minor G allele) predicts mucocutaneous involvement and older age at disease onset among Asians, C3 consumption in boys, and higher anti-dsDNA antibody titers in African/Caribbean participants. Functional studies link genetic variation with impact as rs2302267/n.−20T >G, which may protect from leukopenia through reduced *TLR7* promoter activity, rs3853839/c.∗881 C >G-3’UTR increases *TLR7* mRNA stability, thereby enhancing gene expression and IFN signaling.

Taken together, genetic variation associates with disease risk, organ involvement and outcomes but signatures are complex and incompletely understood. Considering large extended haplotypes and functional impacts may revolutionize our understanding of SLE and allow risk prediction and individualized care [15].

### Genetic Risk Scores To Predict Disease Outcomes

Over recent years, innovative approaches have been applied, identifying previously not appreciated SLE-associated variation [86]. A recent multi-ancestral study including participants of East-Asian, European and admixed American ancestries performed multitrait analyses using summary statistics from 13 other autoimmune/inflammatory diseases to confirm known and identify additional SLE risk loci [86]. Because of the possible cumulative impact of common variants, genetic variability in SLE has been suggested as a tool to predict disease outcomes and organ involvement through genetic risk scores (GRS), polygenic risk scores (PRS), or genome/gene specific alternate allele counts (AAC). GRS aggregate effects of specific gene variants previously associated with SLE [87]. PRS consider the cumulative effect of genetic variants across the genome, each contributing to a small extent to the overall risk of developing a disease. PRS are derived from GWAS and can include thousands of variants, not all of them necessarily previously associated with SLE [87]. AAC consider all genetic variants across a region (not only those previously associated with SLE) [88] **(**Table 2**)**.

**Table 2:** Genetic risk scores in jSLE

Reference	Score	Gene	Variant (rsID) and genomic localisation
Sayadi et al. 2025 [19]	Polygenic risk score created from 37 SLE genome-wide association study single nucleotide variants (SNVs)	*ABHD6-PXK*	rs64459723, rs6445975
		*BACH2*	rs597325
		*BANK1*	rs10028805
		*CD44*	rs2732552
		*CD45*	rs34889541/rs16843520
		*CLEC16A-CIITA-SOCS1*	rs9652601
		*FCGR2A*	rs1801274
		*GRB2*	rs930297
		*ICAM1-ICAM4-ICAM5*	rs3093030
		*IFIH1*	rs10930046, rs2111485
		*IKZF1*	rs4917014
		*IKZF3*	rs2941509
		*IL10*	rs3024505
		*IL21*	rs907715
		*ITGAM-ITGAX*	rs34572943
		*LBH*	rs7579944
		*LYN*	rs7829816
		*NCF2*	rs17849502
		*PRDM1-ATG5*	rs6568431
		*PTPN22*	rs2476601
		*RNASEH2C*	rs1308020/rs489574
		*RNF114*	rs11697848
		*SH2B3-ATXN2*	rs10774625
		*SLC15A4*	rs1059312
		*SPRED2*	rs6740462
		*TCF7-SKP1*	rs7726414
		*TMEM39A*	rs1132200
		*TNFAIP3*	rs6932056
		*TNIP1*	rs7708392/rs6889239
		*TNPO3-IRF5*	rs2070197
		*TYK2*	rs2304256
		*UBE2L3-YDJC-HIC2*	rs7444
		*UHRF1BP1*	rs11755393
		*WDFY4*	rs877819
		*ZPF90*	rs1170426/rs1170427
Misztal et al. 2023 [14]	Additive polygenic risk scores (PRS) for 7 HLA and 39 non-HLA associations.	*ATG5*	rs2299864
		*BANK1*	rs4426778
		*BLK*	rs2736336
		*C7orf72-IKZF1*	rs4917014
		*CLEC16A*	rs9652601
		*DGKQ*	rs4690229
		*ERBB2-IKZF3*	rs4252665
		*ETS1*	rs12575600
		*FAM86B3P*	rs2980512
		*FCGR2A*	rs12129787
		*GRB2*	rs930297
		*GTF2IRD1-GTF2I*	rs73366469
		*HLA Cluster*	HLA_B*0801, HLA_B*1801, HLA_C*0401, HLA_DQA1*0102, HLA_DQA1*0401, HLA_DQB1*0302, HLA_DRB1*0301
		*IFIH1*	rs2111485
		*IL10*	rs3122605
		*IL12A*	rs564976
		*IRF5-TNPO3*	rs12706861
		*IRF7*	rs112006329
		*IRF8*	rs11117431
		*ITGAM*	rs1143679
		*JAZF1*	rs10245867
		*LRRC16A*	rs35789010
		*MSRA*	rs7819602
		*NMNAT2*	rs17484292
		*OLIG3-LOC100130476*	rs2327832
		*PKIA-ZC2HC1A*	rs1966115
		*PTPN22*	rs2476601
		*PTTG1-MIR146A*	rs2431697
		*PXK*	rs180977001
		*SLC15A4*	rs11059927
		*SLC17A4*	rs36014129
		*STAT4*	rs7582694
		*TMEM39A-TIMMDC1*	rs1131265
		*TNFAIP3*	rs77000060
		*TNFSF4-LOC100506023*	rs2205960
		*TNIP1*	rs6889239
		*TYK2*	rs34725611
		*UBE2L3*	rs131658
		*UHRF1BP1-DEF6*	rs9462027
		*WDFY4*	rs2663054
Dominguez et al. 2021 [89]	Genetic risk counting scores (GRCS), genetic risk weighted scores (GRWS,) genetic risk standardized counting score (GRSCS), and genetic risk standardized weighted score (GRSWS). All scores were calculated separately for HLA and non-HLA SNPs and analyzed in the total population.	*ATG5*	rs6568431
		*8p23.1*	rs6601327, Chr:8p23.1
		*BANK1*	rs10516487, rs17266594, rs3733197, rs4522865
		*BLK*	rs2736340, rs2254546, rs2618476, rs2248932, rs7812879, rs2618479, rs10903340, rs13277113
		*C8orf12*	rs7836059
		*C6orf10*	rs7775397
		*ETS1*	rs1128334, rs6590330
		*FAM167A*	rs12680762
		*FCGR2A*	rs1801274
		*HIC2-UBE2L3*	rs463426, rs131654
		*HIP1*	rs6964720
		*HLA(CFB)*	rs1270942
		*HLA(MSH5)*	rs3131379
		*HLA-DQB1-DQA2*	rs1794282
		*HLA-DQA1*	rs2187668, rs9271366
		*HLA-DQA2*	rs3997854
		*HLA-DQB1*	rs9273349, rs1063355
		*HLA-DQB1/HLA-DQA2*	rs9275572/rs2856717, rs9275328/rs9275332
		*HLA-DRA*	rs7192, rs9501626, rs9268979/rs9268877, rs6903608, rs9268880
		*HLA-DRB1*	rs9270984/rs9270986, rs9271100, rs660895
		*IKZF1*	rs4917014
		*IRF5*	rs4728142
		*IRF5/TNPO3*	rs10488631, rs729302, rs10279821, rs12537284
		*ITGAM*	rs9888739, rs4548893, rs1143678
		*ITGAM/ITGAX*	rs11574637
		*KIAA1542*	rs4963128
		*LRRC18-WDFY4*	rs1913517
		*LYN*	rs7829816, rs2667978
		*Multiple genes*	rs1167796, rs4639966, rs7197475
		*NMNAT2*	rs2022013
		*PRDM1-ATG5*	rs548234
		*PTPN22*	rs2476601
		*PTTG1*	rs2431697
		*PXK*	rs6445975
		*RASGRP3*	rs13385731
		*RNF114*	rs11697848
		*SLC15A4*	rs10847697, rs1385374
		*STAT4*	rs7601754, rs3821236, rs7574865, rs10168266, rs10931481
		*TET3*	rs6705628
		*TNFIAP3*	rs2230926, rs5029939, rs7749323, rs3757173
		*TNFSF4*	rs1234315, rs2205960, rs704840, s10798269
		*TNIP1*	rs10036748
		*TNPO3*	rs12531711
		*TNXB*	rs3130342
		*UBE2L3*	rs5754217
		*UHRF1BP1*	rs3734266, rs205284, rs3734261, rs7764921, rs13205210
		*WDFY4*	rs7097397, rs877819
		*XKR6*	rs4240671, rs6985109, rs11783247, rs6984496
Tang et al. 2023 [90]	Additive polygenic risk scores (PRS) for eGFR, SLE HLA and Non-HLA PRSs, using genome-wide significant SNPs from the largest GWAS for eGFR and SLE in Europeans. SLE-PRSs included 39 non-HLA SNPs and seven HLA alleles weighted by log odds ratios from SLE GWAS.	*PTPN22*	rs2476601,Chr.:1p13
		*ATG5*	rs2299864,Chr.:6q21
		*BANK1*	rs4426778,Chr.:4q24
		*BLK*	rs2736336,Chr.:8p23
		*C7orf72-IKZF1*	rs4917014,Chr.:7p12
		*CLEC16A*	rs9652601,Chr.:16p13
		*DGKQ*	rs4690229,Chr.:4p16
		*ERBB2-IKZF3*	rs4252665,Chr.:17q12
		*ETS1*	rs12575600,Chr.:11q24
		*FAM86B3P*	rs2980512,Chr.:8p23
		*FCGR2A*	rs12129787,Chr.:1q23
		*GRB2*	rs930297,Chr.:17q25
		*GTF2IRD1-GTF2I*	rs73366469,Chr.:7q11
		*HLA Cluster*	*HLA_B_0801*,* HLA_B_1801*,* HLA_C_0401*,* HLA_DQA1_0102*,* HLA_DQA1_0401*,* HLA_DQB1_0302*,* HLA_DRB1_0301*
		*IFIH1*	rs7582694,Chr.:2q32
		*IL10*	rs3122605,Chr.:1q32
		*IL12A*	rs564976,Chr.:3q25
		*IRF5-TNPO3*	rs12706861,Chr.:7q32
		*IRF7*	rs2663054,Chr.:10q11
		*IRF8*	rs11117431,Chr.:16q24
		*ITGAM*	rs1143679,Chr.:16p11
		*JAZF1*	rs10245867,Chr.:7p15
		*LRRC16A*	rs35789010,Chr.:6p22
		*MSRA*	rs7819602,Chr.:8p23
		*NMNAT2*	rs17484292,Chr.:1q25
		*OLIG3-LOC100130476*	rs2327832,Chr.:6q23
		*PKIA-ZC2HC1A*	rs1966115,Chr.:8q21
		*PTTG1-MIR146A*	rs2431697,Chr.:5q33
		*PXK*	rs180977001,Chr.:3p14
		*SLC15A4*	rs11059927,Chr.:12q24
		*SLC17A4*	rs36014129,Chr.:6p22
		*STAT4*	rs7582694
		*TMEM39A-TIMMDC1*	rs1131265,Chr.:3q13
		*TNFAIP3*	rs77000060
		*TNFSF4-LOC100506023*	rs2205960,Chr.:1q25
		*TNIP1*	rs6889239,Chr.:5q33
		*TYK2*	rs34725611,Chr.:19p13
		*UBE2L3*	rs131658,Chr.:22q11
		*UHRF1BP1-DEF6*	rs9462027,Chr.:6p21
		*WDFY4*	rs2663054,Chr.:10q11
Webber et al. 2020 [83]	Additive polygenic risk scores (PRS) for 7 HLA and 39 non-HLA associations.	As in [90]	As in [90]
Natoli et al. 2025 [88]	For each participant, genetic risk burden scores were calculated by multiplying alternate allele count by the assigned severity annotation (MODIFIER and LOW = 1, MODERATE = 2, and HIGH = 3). Mutation burden scores were then aggregated at the gene level by summing scores across all variants within a gene for each participant. This resulted in a matrix of 62 Gene-level alternate allele scores (GAAS) for all patients	*AC020594.5*	N/A
		*ACP5*	N/A
		*ACTA2*	N/A
		*ADAR*	N/A
		*AFF1*	N/A
		*AP4B1-AS1*	N/A
		*ATG5*	N/A
		*BANK1*	N/A
		*BLK*	N/A
		*C1R*	N/A
		*C1S*	N/A
		*C2*	N/A
		*C3*	N/A
		*C4orf36*	N/A
		*CFB*	N/A
		*CSK*	N/A
		*CTLA4*	N/A
		*DNASE1*	N/A
		*DNASE1L3*	N/A
		*ETS1*	N/A
		*FAM167A-BLK*	N/A
		*FAS*	N/A
		*FASLG*	N/A
		*GPR108*	N/A
		*GPX3*	N/A
		*GUCY1B2*	N/A
		*IFIH1*	N/A
		*IKZF1*	N/A
		*IL10*	N/A
		*IL21*	N/A
		*IRAK1*	N/A
		*IRF5*	N/A
		*IRF6*	N/A
		*IRF7*	N/A
		*IRF8*	N/A
		*ITGAM*	N/A
		*KAT5*	N/A
		*LYN*	N/A
		*MECP2*	N/A
		*MIR4513*	N/A
		*MSH5*	N/A
		*MSH5-SAPCD1*	N/A
		*NCF1*	N/A
		*NCF2*	N/A
		*PEPD*	N/A
		*PHRF1*	N/A
		*PRDM1*	N/A
		*PRDX2*	N/A
		*PRKCB*	N/A
		*PTPN22*	N/A
		*PXK*	N/A
		*RASGRP3*	N/A
		*RNA5SP29*	N/A
		*RNASEH2A*	N/A
		*RNASEH2B*	N/A
		*RNASEH2B-AS1*	N/A
		*RNASEH2C*	N/A
		*RP1-134E15.3*	N/A
		*RP11-1007G5.2*	N/A
		*RP11-148O21.2*	N/A
		*RP11-148O21.4*	N/A
		*RP11-21K12.2*	N/A
		*RP11-356I2.4*	N/A
		*RP11-399O19.9*	N/A
		*RP11-802O23.3*	N/A
		*RP5-1073O3.2*	N/A
		*SAMHD1*	N/A
		*SAPCD1*	N/A
		*SLC15A4*	N/A
		*SLX4*	N/A
		*STAT4*	N/A
		*TLDC2*	N/A
		*TLR7*	N/A
		*TMEM173*	N/A
		*TNFAIP3*	N/A
		*TNFSF4*	N/A
		*TNIP1*	N/A
		*TRAP1*	N/A
		*TREX1*	N/A
		*TYK2*	N/A
		*UBE2L3*	N/A
		*ZBTB12*	N/A
		*ZNF627*	N/A
Lewandowski et al. 2025 [76]	39 common SLE risk variants (seven in human leucocyte antigen (HLA) region). A PRS was calculated using PRSice23 for each individual participant based on the sum of the product of the natural logarithm of the OR for each association and the number of alleles present in each individual at each risk locus.	*ATG5*	rs2299864
		*BANK1*	rs4426778
		*BLK*	rs2736336
		*CLEC16A*	rs9652601
		*DGKQ*	rs4690229
		*ETS1*	rs12575600
		*FAM86BP3*	rs2980512
		*GRB2*	rs930297
		*GTF2IRD1-GTF2I*	rs73366469
		*HLA Cluster*	*HLA Allele sB_08_01*,* B_18_01*,* C_04_01*,* DQA1_01_02*,* DQA1_04_01*,* DQB1_03_02*
		*IFIH1*	rs2111485
		*IL10*	rs3122605
		*IL12A*	rs564976
		*IRF-TNPO3*	rs12706861
		*IRF7*	rs112006329
		*IRF8*	rs11117431
		*ITGAM*	rs1143679
		*LRRC16A*	rs35789010
		*MSRA*	rs7819602
		*NMNAT2*	rs17484292
		*PKIA-ZC2HC1A*	rs1966115
		*PTPN22*	rs2476601
		*PTTG1-MIR146A*	rs2431697
		*PXK*	rs180977001
		*SLC15A4*	rs11059927
		*SLC17A4*	rs36014129
		*STAT4*	rs7582694
		*TMEM39A-TIMMDC1*	rs1131265
		*TNFAIP3*	rs77000060
		*TNFSF4*	rs2205960
		*TNIP1*	rs6889239
		*TYK2*	rs34725611
		*UBE2L3*	rs131658
		*WDFY4*	rs2663054

SNVs: single nucleotide variants; PRS: polygenic risk scores; HLA: human leucocyte antigen; GRCS: genetic risk counting scores; GRWS: genetic risk weighted scores; GRSCS: genetic risk standardized counting score; GRSWS: genetic risk standardized weighted score; eGFR: estimated glomerular filtration rate; GWAS: Genome-Wide Association Study; GAAS: Gene-level alternate allele scores; OR: odd ratio.

Several versions of GRS have been suggested, promising benefit for future patient stratification. In agreement with previous studies applying very small panels of SLE-associated polymorphisms [77], Dominguez et al.. reported an inverse correlation of non-HLA GRS and age at disease onset, while HLA GRS correlated with an older age at diagnosis [89]. Webber et al. observed an association between the ‘genetic load’ of SLE, estimated by HLA and non-HLA GRS, and the risk of LN. The effects of non-HLA and HLA GRS on LN were greater in jSLE when compared to aSLE, although this did not reach statistical significance [83]. Misztal et al., using non-HLA (39 variants) and HLA (7 alleles identified in Europeans with SLE) GRS approaches, suggested that jSLE patients carrying rare deleterious variants (monogenic SLE) do not have higher or lower GRS than non-carriers. This supports the hypothesis that early-onset monogenic jSLE is, indeed, due to rare damaging mutations that do not require additional ‘impacts’ to result in disease [14].

Generating PRS, Tang et al. identified associations between both HLA and non-HLA scores and reduced mean estimated glomerular filtration rates, an approximation of renal function [90]. The authors concluded that PRS may be a powerful tool for LN prediction and monitoring [90]. Sayadi et al. demonstrated that patients with genetically complex jSLE or aSLE had increased PRS when compared to healthy participants. Conversely, PRS in patients with jSLE caused by rare damaging variants (monogenic SLE) were comparable to controls. Notably, aSLE patients with rare variants exhibited PRS comparable to SLE patients without rare variants, suggesting that aSLE-associated rare variants either require a second allele (recessive trait) or additional cumulative genetic risk (risk alleles) [19].

In a recent study, we calculated AAC across a sequencing panel (including SLE-associated genes) and individual genes. We, across patients with genetically complex jSLE, found an inverse correlation between genetic variability and age at disease onset that was primarily driven by South Asian patients [88]. Levandowski et al. reported similar associations when only considering previously reported SLE-associated common and rare variants [76]. Moreover, in the UK JSLE cohort, genetic variability at several SLE-associated genes (namely *ACP5*,* ITGAM*,* LYN*,* TNFAIP3*) associated with high disease activity in pediatric BILAG organ domains (e.g., kidney disease and *ACP5*,* ITGAM*,* LYN*,* TNFAIP3*), suggesting exploitation of this approach to predict organ involvement/outcomes [88].

While recent reports promise clinical implementation, several limitations remain. The majority of GWAS used to build PRS/GRS have been conducted in populations of European ancestry, making the derived scores less accurate in non-European groups [74]. Lack of ancestral diversity across studies raises concerns about equity in genomic medicine and underscores the urgent need to include underrepresented populations [91, 92]. Moreover, despite their recognized role in the etiology of multifactorial diseases, current PRS/GRS models fail to incorporate gene: environment interactions [7]. Environmental exposure, lifestyle, and resulting epigenetic signatures affect the genetic architecture and are not sufficiently captured in current models [87, 93]. Lastly, the unresolved issue of ‘missing heritability’ remains as a substantial portion of disease that cannot yet be explained by currently known variants. Closing this gap will require larger, more diverse genome-wide sequencing initiatives, including all ancestries and age groups, the integration of common and rare variants with clinical information, structural variants, and potentially non-additive genetic effects [7, 94, 95].

## Improved Treatments and Strategies

An improved understanding of the molecular pathophysiology of SLE has improved our ability to offer personalized and target-directed treatments. Indeed, recent years have been marked by unprecedented advances in the therapeutic landscape of SLE, including the approval of the first two biologic agents, belimumab and anifrolumab [96, 97], and the emergence of chimeric antigen receptor (CAR)-T cell therapy [98], an innovative approach that is transforming the prognosis of lupus patients with severe and treatment-refractory disease. Therapeutic strategies in jSLE are primarily guided by data from aSLE studies, but an expanding body of pediatric-specific data are reinforcing the rationale for their use in children. Medications currently used (within and off-label) for the treatment of jSLE and treatments under investigation in clinical trials in the pediatric population are provided in Tables 3 and 4.

**Table 3:** DMARDs available for juvenile-onset systemic lupus erythematosus

Therapeutic compound	Target	Route	Regulatory Approval Status	Main indications
Hydroxychloroquine	Anti-malarial TLR7/3/9 antagonist	Oral	Off-label use	Recommended in all SLE patients, unless contraindicated
Mycophenolate mofetil	Inosine monophosphate dehydrogenase inhibitor	Oral	Off-label use	Lack of response to hydroxychloroquine (+/- GCs) or inability to taper GCs; induction and maintenance phases in LN
Cyclophosphamide	Alkylating agent	IV	Off-label use	Low-dose regimen in induction of LN; high-dose regimen in refractory, organ-threatening or life-threatening disease, especially in LN patients with high risk for kidney failure, severe NPSLE and autoimmune thrombocytopenia
Azathioprine	Purine antagonist	Oral	Off-label use	Lack of response to hydroxychloroquine (+/- GCs) or inability to taper GCs; maintenance phase in LN and severe autoimmune thrombocytopenia
Methotrexate	Dihydrofolate reductase inhibitor	Oral/SC	Off-label use	Lack of response to hydroxychloroquine (+/- GCs) or inability to taper GCs; especially if joint involvement
Rituximab	CD20 antibody	IV	Off-label use	Refractory, organ-threatening or life-threatening disease, especially in case of renal of haematological involvement
Belimumab	BLyS inhibitor	IV/SC	FDA/EMA-approved for paediatric SLE and LN ≥ 5 years	Lack of response to hydroxychloroquine (+/- GCs) or inability to taper GCs; combination therapy in the induction phase of LN (MMF or low-dose CYC + GCs)
Anifrolumab	IFNα receptor antibody	IV	FDA/EMA-approved for adult SLE; not yet approved in jSLE.	Lack of response to hydroxychloroquine (+/- GCs) or inability to taper GCs, especially if cutaneous and musculoskeletal involvement
Cyclosporine	Calcineurin inhibitor	Oral	Off-label use	Combination therapy in the induction phase of LN (MMF + GCs); maintenance treatment of severe autoimmune thrombocytopenia
Tacrolimus	Calcineurin inhibitor	Oral	Off-label use	Combination therapy in the induction phase of LN (MMF + GCs)
Voclosporin	Calcineurin inhibitor	Oral	FDA/EMA-approved for adult SLE; not yet approved in jSLE.	Combination therapy in the induction phase of LN (MMF + GCs)

CYC, cyclophosphamide; DMARDs, Disease-Modifying Antirheumatic Drugs; EMA, European Medicines Agency; FDA, Food and Drug Administration; GCs, glucocorticoids; IFN, interferon; intravenous; jSLE, juvenile systemic lupus erythematosus; LN, lupus nephritis; MMF, mycophenolate mofetil; NPSLE, neuropsychiatric systemic lupus erythematosus; SC, subcutaneous; SLE, systemic lupus erythematosus; TLR, toll-like receptor

**Table 4:** Treatments under investigation in randomized clinical trials for juvenile-onset systemic lupus erythematosus

Therapeutic compound	Target/mechanism of action	Age group (population)	Current trial status	Trial phase	Study identifier	Key outcomes/notes	Reference
Belimumab (IV)	Fully human monoclonal antibody binding soluble B-lymphocyte stimulator (BLyS)	≥ 5 years	Completed	Phase 1	NCT05917288	-	
		≥ 5 years	Completed, with results	Phase 4	NCT04908865	67 patients included; 66.7% achieved ≥ 4-point SELENA-SLEDAI reduction at Week 52 (primary endpoint); no AESIs or new safety concerns	[99]
		≥ 5 years	Active, not recruiting	Phase 2	NCT01649765	-	
		≥ 14 years	Unknown status	Phase 3	NCT05863936	-	
Belimumab (SC)		≥ 5 years	Active, not recruiting, with results	Phase 2	NCT04179032	25 jSLE patients included, SC belimumab exposure consistent with adult SC 200 mg/week regimen based on population PK modeling. Mean concentrations higher in ≥ 50 kg vs. ≥ 30–<50 kg group; safety profile consistent with prior studies, no new safety concerns.	[100]
Obinutuzumab	Humanized type II anti-CD20 monoclonal antibody inducing enhanced B-cell depletion	≥ 14 years	Recruiting	Phase 3	NCT04702256	RCT comparing oral corticosteroids plus MMF vs. obinutuzumab and MMF (OBILUP)	
		≥ 5 years	Recruiting	Phase 2	NCT05039619	-	
Ocrelizumab	Humanized monoclonal antibody targeting CD20, inducing B-cell depletion	≥ 16 years	Terminated	Phase 2	NCT00539838	The study was terminated prematurely when the decision was made that ocrelizumab was not likely to benefit this patient population.	
		≥ 16 years	Terminated	Phase 3	NCT00626197	Study was terminated due to an imbalance of serious and opportunistic infections in the ocrelizumab treated patients versus the placebo arm.	
Anti-CD19 Universal CAR T Cells	Allogeneic CAR-engineered T cells targeting CD19, inducing B-cell depletion	≥ 5 years	Recruiting	Phase 1	NCT06691152	-	
Anti-CD19 NEX-T CAR T Cells	Autologous CAR-engineered T cells targeting CD19, inducing antigen-specific B-cell depletion (next-generation construct)	≥ 16 years	Recruiting	Phase 2	NCT07015983	-	
Anti-CD19 CAR T Cells	Autologous CAR-engineered T cells targeting CD19, inducing antigen-specific B-cell depletion	≥ 3 years	Recruiting	Phase 1/2	NCT06585514	-	
		≥ 14 years	Active, recruiting	Phase 1/2	NCT06675422	-	
		≥ 12 years	Not yet recruiting	Phase 2	NCT07053800	-	
		≥ 5 years	Active, not yet recruiting	Phase 1	NCT06222853	-	
		≥ 12 years	Recruiting	Phase 1/2	NCT06839976	-	
		≥ 6 years	Recruiting	Phase 3	NCT06904729		
CNTY-101	Allogeneic CAR-engineered NK cells targeting CD19, inducing B-cell depletion with enhanced persistence and immune evasion.	≥ 17 years	Recruiting	Phase 1	NCT06255028	-	
Anti-BCMA/CD70 CAR-T Cells	Dual-target CAR-T cells recognizing BCMA and CD70	≥ 5 years	Recruiting	Phase 1	NCT06934447	-	
Anti-CD19/BCMA CART Cells	Dual CAR-T cells simultaneously targeting CD19⁺ B cells and BCMA⁺ plasmablasts/plasma cells	≥ 6 years	Recruiting	Phase 1/2	NCT06947460	-	
		Children (age range not specified)	Unknown	Early Phase 1	NCT05085418	-	
Umbilical Cord Blood CD19-BCMA CART Cell Therapy	Dual CAR-T cells derived from umbilical cord blood that target CD19⁺ B cells and BCMA⁺ plasmablasts/plasma cells	≥ 6 years	Not yet recruiting	Phase 1/2	NCT06947473	-	
Descartes-08	Transient mRNA-engineered anti-BCMA CAR-T cells that selectively target and eliminate autoreactive plasma cells and plasmablasts	≥ 12 years	Recruiting	Phase 1/2	NCT07089121	-	
FT819	Engineered iPSC-derived T cells expressing CD19-CAR mediate antigen-specific cytotoxicity without TCR signaling, enabling off-the-shelf allogeneic use.	≥ 12 years	Recruiting	Phase 1	NCT06308978	-	
Blinatumomab	Bispecific T-cell engager antibody targeting CD19 and CD3 inducing T-cell-mediated B-cell lysis	≥ 5 years	Recruiting	Phase 1	NCT06789107	-	
Telitacicept	Fully human TACI-Fc fusion protein targeting B lymphocyte stimulator (BLyS) and a proliferating-inducing ligand (APRIL)	≥ 12 years	Recruiting	Phase 3	NCT06456567	-	
		≥ 12 years	Recruiting	Phase 1	NCT05687526	-	
		≥ 12 years	Active, not recruiting	Phase 3	NCT05306574	-	
Atacicept	Recombinant fusion protein (TACI-Ig) blocking B-lymphocyte stimulator (BLyS) and a proliferation-inducing ligand (APRIL)	≥ 16 years	Completed, with results	Phase 2/3	NCT00624338	461 patients randomized; primary endpoint (flare rate) not different for atacicept 75 mg vs. placebo; atacicept 150 mg (arm discontinued after 2 deaths) showed reduced flare rate (OR 0.48, *p* = 0.002) and longer time to first flare (HR 0.56, *p* = 0.009). AEs mostly mild/moderate.	[101]
		≥ 16 years	Terminated	Phase 2/3	NCT00573157	The study was terminated due to unanticipated safety issues	
Ianalumab	Fully human monoclonal antibody targeting the BAFF receptor (BAFF-R)	≥ 12 years	Recruiting	Phase 3	NCT06133972	-	
		≥ 12 years	Recruiting	Phase 3	NCT05624749	-	
		≥ 12 years	Recruiting	Phase 3	NCT05639114	-	
Abetimus sodium (LJP 394)	Induces B-cell tolerance by cross-linking anti-dsDNA B-cell receptors, leading to selective suppression of autoreactive B cells.	≥ 12 years	Terminated	Phase 1/2	NCT00089804	Interim efficacy analysis indicated it would be futile to continue study.	
		≥ 12 years	Completed, with results	Phase 3	NCT00035308	317 LN patients included; renal flare not significantly delayed, but flare rate numerically lower (12% vs. 16%). Anti-dsDNA significantly reduced (*P* < 0.0001) with associated C3 increase. More patients achieved ≥ 50% proteinuria reduction at 1 year (nominal *P* = 0.047). Treatment well tolerated.	[102]
Dapirolizumab pegol	PEGylated anti-CD40L (CD154) Fab’ fragment targeting CD40-CD40L (co-stimulation blocker)	≥ 16 years	Recruiting	Phase 3	NCT06617325	-	
		≥ 16 years	Enrolling by invitation	Phase 3	NCT04976322	-	
		≥ 16 years	Completed, with results	Phase 3	NCT04294667	315 patients randomized (2:1); primary endpoint met: BICLA response at Week 48 in 49.5% (DZP + SOC) vs. 34.6% (PBO + SOC; *p* = 0.011). SRI-4 response 60.1% vs. 41.1%; severe BILAG flares 11.6% vs. 23.4%. 72.4% vs. 52.9% achieved GC taper ≤ 7.5 mg/day. TEAEs: 82.6% vs. 75.0%; serious TEAEs lower with DZP (9.9% vs. 14.8%); no unexpected safety issues.	[103]
Anifrolumab	Fully human monoclonal antibody targeting type I interferon receptor subunit 1 (IFNAR1)	≥ 5 years	Recruiting	Phase 3	NCT05835310	-	
Voclosporin	Next-generation calcineurin inhibitor	≥ 5 years	Enrolling by invitation	Phase 3	NCT05962788	-	
		≥ 5 years	Recruiting	Phase 3	NCT05288855	-	
Tacrolimus	Calcineurin inhibitor	≥ 14 years	Completed	Phase 3	NCT01328834		-
		≥ 14 years	Completed, with results	Phase 3	NCT00615173	81 LN patients randomized (tacrolimus *n* = 42 vs. IV cyclophosphamide *n* = 39). Complete remission numerically higher with tacrolimus (52.4% vs. 38.5%, NS). Proteinuria reduction significantly faster with tacrolimus at 1 month (1.76 vs. 2.40 g/day, *P* = 0.02). Response rate high in both groups (90.5% vs. 82.1%). Safety profile more favorable with tacrolimus (fewer leukopenia and GI events).	[104]
Dapirolizumab pegol	PEGylated anti-CD40L (CD154) Fab’ fragment targeting CD40-CD40L (co-stimulation blocker)	≥ 16 years	Recruiting	Phase 3	NCT06617325	-	
		≥ 16 years	Enrolling by invitation	Phase 3	NCT04976322	-	
		≥ 16 years	Completed, with results	Phase 3	NCT04294667	315 patients randomized (2:1); primary endpoint met: BICLA response at Week 48 in 49.5% (DZP + SOC) vs. 34.6% (PBO + SOC; *p* = 0.011). SRI-4 response 60.1% vs. 41.1%; severe BILAG flares 11.6% vs. 23.4%. 72.4% vs. 52.9% achieved GC taper ≤ 7.5 mg/day. TEAEs: 82.6% vs. 75.0%; serious TEAEs lower with DZP (9.9% vs. 14.8%); no unexpected safety issues.	[103]
Ustekinumab	Fully human monoclonal antibody targeting the p40 subunit shared by IL-12 and IL-23	≥ 16 years	Terrminated, with results	Phase 3	NCT03517722	516 patients randomized (3:2); primary endpoint not met: SRI-4 response at Week 52: 44% (ustekinumab) vs. 56% (placebo). No difference in major secondary endpoints or flare rates; safety profile consistent with prior experience.	
Abatacept	Fusion protein blocking T-cell costimulation via CTLA-4–mediated CD80/CD86 inhibition.	≥ 16 years	Completed	Phase 2	NCT00774852	134 patients with active lupus nephritis included; abatacept added to low-dose CYC followed by AZA did not improve complete response rates at week 24 (33% vs. 31% placebo) or week 52; safety profile comparable between groups.	[105]
Abacavir/lamivudine	Nucleoside reverse transcriptase inhibitors	≥ 12 years	Not yet recruiting	Phase 2	NCT06356740	-	
Artesunate	Semisynthetic artemisinin derivative inhibiting STAT3, modulating Th17 differentiation and type I interferon–linked inflammatory signaling.	≥ 14 years	Uknown status	Phase 4	NCT03214731	-	
Lymphodepleting autologous haematopoietic cell transplantation	Immune “reset” with naïve/Treg repopulation and sustained suppression of type I IFN and autoantibody activity.	≥ 15 years	Completed, with results	Phase 2	NCT00076752	8 patients conditioning with CYC, fludarabine, rituximab. 5/8 achieved complete response (4 sustained ≥ 11 years); 1 partial response, 2 early deaths (infection, MOF). AHSCT induced profound lymphodepletion, Treg expansion, IFN gene signature suppression, and sustained anti-dsDNA clearance	[102]
Autologous Stem Cell Transplant	Depletion of CD3/CD19 in an autologous stem cell transplant	≥ 5 years	Recruiting	Phase 2	NCT05029336	-	
Mesenchymal Stem Cells Transplantation	Immunomodulation via paracrine suppression of T/B cells and induction of regulatory pathways.	≥ 15 years	Completed, with results	Phase 1/2	NCT00698191	404 patients with autoimmune diseases (including 178 SLE) included. 5-year survival 90.4%, 8-year 88.9%. Infection rate 29.5% (serious 12.9%), malignancy 1.2%, and transplantation-related mortality 0.2%.	[106]
Allogeneic Amniotic Mesenchymal Stem Cell Therapy	Immunomodulation via paracrine suppression of T/B cells and induction of regulatory pathways.	≥ 14 years	Completed	Phase 1	NCT04318600	-	
Allogeneic Umbilical Cord-derived Mesenchymal Stem Cell	Modulate immune tolerance by suppressing pathogenic T/B-cell responses, enhancing regulatory T cells, and dampening pro-inflammatory cytokine and dendritic cell activation.	≥ 16 years	Completed, with results	Phase 2	NCT01539902	18 LN patients included; remission rates similar between MSC and placebo (75% vs. 83%), with no significant differences in proteinuria, renal function, complement or disease activity scores. Safety signals emerged including 2 serious infections and 1 death in the MSC group.	[107]

Therapeutic agents included in this table were identified through a systematic search of interventional randomized clinical trials registered on ClinicalTrials.gov, updated to 16 November 2025, restricted to studies enrolling also patients with juvenile-onset systemic lupus erythematosus younger than 18 years. Abbreviations: AESI, Adverse Event of Special Interest; AHSCT, Autologous Hematopoietic Stem Cell Transplantation; APRIL, A Proliferation-Inducing Ligand; AZA, Azathioprine; BAFF, B-cell Activating Factor; BAFF-R, BAFF Receptor; BCMA, B-cell Maturation Antigen; BICLA, BILAG-Based Composite Lupus Assessment; BILAG, British Isles Lupus Assessment Group; BLyS, B-Lymphocyte Stimulator; CAR-T, Chimeric Antigen Receptor T cells; CD, Cluster of Differentiation; CR, Complete Response; CYC, Cyclophosphamide; Anti-dsDNA, Anti-Double-Stranded DNA; GC, Glucocorticoid; GI, Gastrointestinal; HR, Hazard Ratio; IFN, Interferon; IFNAR1, Interferon-α/β Receptor Subunit 1; IL, Interleukin; IV, Intravenous; MMF, Mycophenolate Mofetil; MOF, Multi-Organ Failure; MSC, Mesenchymal Stem Cell; NK, Natural Killer; OR, Odds Ratio; PBO, Placebo; PK, Pharmacokinetics; RCT, Randomized Controlled Trial; SC, Subcutaneous; SLE, Systemic Lupus Erythematosus; SLEDAI, SLE Disease Activity Index; SOC, Standard of Care; STAT3, Signal Transducer and Activator of Transcription 3; SRI-4, Systemic Lupus Responder Index-4; TACI, Transmembrane Activator and CAML Interactor; TCR, T-Cell Receptor; TEAE, Treatment-Emergent Adverse Event; Th17, T-helper 17 cells

### Biological and Targeted Therapies

#### B cell targeting treatments

Belimumab, a monoclonal antibody targeting B lymphocyte stimulator (BLyS), is the only biologic disease-modifying antirheumatic drug formally approved for use in jSLE (≥ 5 years). Its safety and efficacy in reducing flares, enabling glucocorticoid tapering, and maintaining disease control have been confirmed in clinical trials, real-world cohorts and long-term extension studies across adult and pediatric cohorts [96, 108–111].

Rituximab, a type I anti-CD20 monoclonal antibody, despite not being formally approved for SLE (it did not meet endpoints in EXPLORER and LUNAR studies), remains a cornerstone of jSLE management [112, 113], and is included in clinical guidelines for refractory SLE [114]. Real-world data support its effectiveness, especially when used early in jSLE [115, 116] but also as add-on treatment in the maintenance phase of patients with serologically active jSLE [117]. In a recent case series, rituximab was used in combination regimens, including with daratumumab, a monoclonal antibody targeting CD38 initially developed for multiple myeloma, to target B cells and long-lived plasma cells in two jSLE patients with refractory LN [118].

Obinutuzumab, a type II anti-CD20 monoclonal antibody, achieves more profound and sustained B cell depletion than type I anti-CD20 antibodies (e.g., rituximab). Obinutuzumab, in addition to standard therapy, demonstrated greater efficacy in achieving complete renal response at week 76 in adults with active LN (46.4% versus 33.1%; 95%CI:2.0–24.8.0.8; *p* = 0.02) [119]. Following this successful RCT, recently updated EULAR recommendations on the management of renal involvement in SLE endorsed the use of obinutuzumab (in combination with MMF) as induction and maintenance treatment [120].

Evidence from paediatric cohorts remains limited to single-centre and observational studies on other nephropathies, including steroid-dependent nephrotic syndrome [121] and steroid- and rituximab-resistant podocytopathies [122], showing a good safety profile and effectiveness. A phase 2 randomized controlled trial (RCT) is currently recruiting pediatric patients with LN (NCT05039619). Bortezomib, a proteasome inhibitor, has shown potential in refractory neuropsychiatric manifestations, although experience in jSLE remains limited to a small case series [123].

One of the most promising recent developments is CD19-directed CAR-T cell therapy, which has shown potential to induce sustained drug-free remission and resolution of major organ involvement in adults with refractory jSLE or aSLE, with acceptable short- and long-term safety profiles [98, 124, 125]. Evidence in pediatric patients is still very limited, although recent case reports suggest responses in adolescents, including recovery from dialysis-dependent LN [126–128]. However, the durability of response and relapse risk remain uncertain. Several clinical trials recruiting pediatric patients are ongoing **(**Table 3**)**. Despite early encouraging results, CAR-T cell therapy remains a highly complex and costly approach, requiring personalized cell manufacturing in specialized centers [98, 129, 130]. Important limitations include the need for an intensive lymphodepleting procedure before CAR-T cell infusion, adding significant toxicity, and short-term immune-mediated toxicity such as cytokine release syndrome and immune effector cell-associated neurotoxicity syndrome (although typically of mild to moderate grade in autoimmune indications) [124]. Use in children raises specific safety considerations, including the need for intense lymphodepletion, increased infection risk, and the unknown long-term impact on immune system development and vaccine responsiveness. In addition, genetic testing may be warranted before treatment (especially in young patients with exceptionally severe phenotypes, who are those most likely to be candidates for this approach) as monogenic lupus or immune dysregulation syndromes could influence both efficacy and safety. Therefore, CAR-T cell therapy should currently be considered only in highly selected cases and ideally within controlled clinical trials [131]. Moreover, given the novelty and perceived transformative potential of this approach, reporting bias favouring successful cases cannot be excluded [98, 129, 130].

In parallel, bispecific T cell engagers (BiTEs) have gained increasing attention as promising immunotherapy in autoimmune/inflammatory diseases, including SLE. These bioengineered antibodies simultaneously bind a T cell (via CD3) and a target B cell (via CD19, CD20, BCMA/biological target B cell maturation antigen, etc.), inducing direct cytotoxic activity without the need for patient-specific cell modification. Compared to CAR-T cell therapies, BiTEs may offer advantages in terms of availability, manufacturing complexity and costs. Nonetheless, the B cell depletion efficacy in tissues appears less profound and sustained when compared to CAR-T cells [132]. The first anti-BCMA bispecific antibody reported in SLE was teclistamab (BCMA×CD3) and demonstrated efficacy in a SLE patient with refractory disease. Clinical trials are currently underway to evaluate mosunetuzumab (CD20×CD3) (NCT05155345) and blinatumomab (CD19×CD3) (NCT06570798) in SLE [133, 134]. Although still in the early stages of investigation, particularly in paediatric populations, BiTEs represent a novel and likely more versatile strategy with the potential to broaden the therapeutic landscape.

Novel B cell-directed therapies include telitacicept, a dual BAFF/APRIL inhibitor recently approved for adult SLE in China. In a phase 2b trial (*n* = 249), telitacicept significantly increased SRI-4 responses at week 48 (up to 75.8% versus 33.9% placebo), with favourable biomarker changes and a safety profile comparable to placebo [135]. Lanalumab, a monoclonal anti-BAFF-R antibody, demonstrated encouraging results in phase 2 trials, with 44% achieving SRI-4 with sustained steroid tapering vs. 9% placebo at week 28, reduced flare rates, and consistent B cell depletion. Benefits were maintained through 52 weeks, with a similar responder rate (40–46%) and no drug-related serious adverse events [136, 137]. These findings support ongoing phase 3 RCTs, including studies enrolling adolescents (NCT06133972, NCT05624749, NCT05639114).

#### Co-stimulatory pathway inhibitors

Dapirolizumab pegol is a PEGylated Fab’ fragment targeting CD40L, developed to inhibit CD40:CD40L-mediated T: B cell co-stimulation. In the phase 2 RISE trial, BICLA responses at week 24 were numerically higher with dapirolizumab pegol versus placebo (44–48% versus 37%), although the primary dose-response endpoint was not achieved (*p* = 0.07) [138]. In the phase 3 PHOENYCS GO program, treatment with dapirolizumab pegol resulted in improvements across global disease activity, organ-specific outcomes, flare reduction and serological markers [139, 140]. Most recently, updated results showed higher BICLA response rates at week 48 (49.5% vs. 34.6%; *p* = 0.011), improved SRI-4 responses (60.1% vs. 41.1%), fewer severe flares (11.6% vs. 23.4%) and higher rates of successful steroid tapering to ≤ 7.5 mg/day (72.4% vs. 52.9%) [103]. Notably, this trial also included adolescents aged ≥ 16 years, and additional RCTs are currently recruiting within this age group (NCT06617325, NCT04976322).

#### Type I IFN-targeting treatment

Anifrolumab, a monoclonal antibody targeting the IFN receptor (IFNAR1), was approved for the treatment of SLE in adults (MUSE, TULIP-1, TULIP-2 trials) [97, 141, 142]. Its long-term efficacy, tolerability, and safety were confirmed by the long-term extension studies TULIP-LTE [143]. Although not yet approved for patients < 18 years, anifrolumab is currently being investigated in pediatric populations, and evidence in children is limited to positive reports in cutaneous lupus [144, 145].

Litifilimab, an anti-BDCA2 monoclonal antibody targeting plasmacytoid dendritic cells, represents a novel strategy to inhibit type I IFN expression without broad immune depletion. In the phase 2 LILAC trial, litifilimab demonstrated modest improvements in joint disease at 24 weeks when compared to placebo, with favorable effects on global and cutaneous activity (e.g., higher SRI-4 and CLASI-A response rates). Safety was comparable to placebo but included a signal for herpesvirus reactivation, underscoring the importance of the ongoing phase 3 RCTs for confirmation of both efficacy and long-term safety (NCT04895241, NCT04961567). Another pDC-targeting antibody, daxdilimab (anti-ILT7), showed mixed phase 2 results in SLE: the primary endpoint was not met, but patients receiving the every-4-weeks regimen achieved higher LLDAS rates than placebo (33.7% vs. 17.2%). Safety was comparable (TEAEs 74.8% vs. 69.0%), supporting further evaluation [146].

### Intracellular signalling-targeting Treatments

#### JAK inhibitors

Because of their ability to block multiple cytokine pathways relevant to SLE pathogenesis, including type I IFN, IL-12, IL-23 and IL-21, JAK inhibitors (JAKi) represent a promising therapeutic approach, particularly for mucocutaneous and articular manifestations. In the past three years, phase 2 (deucravacitinib, upadacitinib/elsubrutinib) and 3 trials (baricitinib) have generated mixed results in aSLE cohorts [147–150]. Among these, baricitinib has been the most extensively studied. While the SLE-BRAVE-I trial met its primary endpoint with higher SRI-4 responses at week 52 for baricitinib 4 mg versus placebo (57% vs. 46%; *p* = 0.016) [147], findings were not confirmed in the subsequent SLE-BRAVE-II trial [148]. Because of discordant efficacy signals, further development of baricitinib in SLE is on hold.

Evidence supporting the use of JAKi in jSLE remains limited, and no randomized controlled trials are currently enrolling. One ongoing mechanistic study is evaluating JAK3 expression in blood and renal tissue of patients between 7 and 16 years with active lupus (NCT04293510). Current data derive mainly from isolated case reports, including a recent case series describing nine girls with jSLE treated with tofacitinib, showing clinical benefit without major safety concerns [151, 152].

#### Calcineurin inhibitors

Calcineurin inhibitors (CNIs) have long been part of the therapeutic arsenal for SLE, particularly in LN. They exert a dual mechanism of action, including the inhibition of T cell activation via the calcium-calmodulin-calcineurin pathway and stabilization of the glomerular podocyte cytoskeleton. Among traditional CNIs, tacrolimus has gained a central role after demonstrating non-inferiority to mycophenolate mofetil (MMF) and intravenous cyclophosphamide in the treatment of LN [153, 154]. However, nephrotoxicity represents a major concern. Over recent years, interest has shifted towards ‘new’ calcineurin inhibitors with improved safety and usability. Voclosporin is a new-generation agent with greater potency and pharmacokinetic stability; its predictable and rapidly cleared metabolite profile eliminates the need for routine therapeutic drug monitoring and contributes to a more favourable safety profile. Voclosporin has been approved for the treatment of LN in adults following the AURORA-1 trial, where voclosporin added to MMF and low-dose glucocorticoids significantly increased complete renal response rates at week 52 (41% versus 23% in placebo group) [155]. Longer-term data from the AURORA-2 extension demonstrated sustained renal response and stable renal function over three years, without new safety signals [156]. Considering evidence from these successful RCTs, voclosporin has been incorporated into major international treatment recommendations for renal SLE, including EULAR, ACR and KDIGO guidelines [120, 157, 158]. While regulatory approval is still lacking for patients < 18 years, ongoing RCTs are evaluating its use in adolescents with LN (NCT02630628, NCT05337124, NCT05288855, NCT05962788).

#### Emerging CNI and B cell-targeted combination approaches

By pairing the rapid antiproteinuric, podocyte-stabilising effects of CNIs with the durable suppression of autoreactive B cell responses, combination therapy strategies are gaining momentum in SLE, especially in case of renal involvement. A recent case series of four adults with refractory proliferative LN treated with voclosporin plus belimumab reported encouraging real-world outcomes, including resolution of proteinuria, successful glucocorticoid withdrawal in all patients, and no serious infections over 8–14 months of follow-up [159]. A subsequent 2025 case report described a young woman with refractory class III/V LN who achieved clinical remission, marked proteinuria reduction and steroid minimisation on belimumab-voclosporin combination approach plus MMF and low-dose prednisone, again without relevant toxicity over nine months [160]. Growing interest has also emerged around more structured evaluations of these approaches: a single-centre retrospective cohort of 33 adults treated with belimumab-tacrolimus demonstrated high retention (>90% at week 52; ~80% at day 1000), a marked increase in LLDAS (from 9.1% at baseline to 64% at week 52), lower flare rates (from 90.9% pre-treatment to 9.1% during the first year and 15.2% over total follow-up), alongside a glucocorticoid-sparing effect (median dose reduced from 8 mg/day to 5 mg/day at week 52, with some patients discontinuing steroids), with only one serious infection [161]. Building on these observations, the phase IV SYNERGY trial (NCT07225387) is currently evaluating belimumab plus voclosporin on a background of MMF and glucocorticoids in aSLE patients with proliferative LN. Evidence in the pediatric population is currently lacking.

### A Paradigm Shift in Lupus Nephritis

Nephritis is the most frequent severe manifestation of jSLE, affecting up to 80% of patients (compared to 20–40% in aSLE), representing a contributor to long-term morbidity and mortality [3]. Despite remission rates >70% within the first year, renal relapses occur in up to 40%, with relapse-free survival dropping to approximately 50% at 10 years [162, 163]. These challenges have promoted a shift from the traditional sequential approach to LN (induction with glucocorticoids plus MMF or cyclophosphamide, followed by maintenance with MMF or azathioprine) [164, 165], to a more potent, multitarget induction strategy in adults with LN. This triple immunomodulating regimen typically consists of glucocorticoids and the combination of MMF and either belimumab or CNIs, or low-dose cyclophosphamide and belimumab. This strategy has been endorsed by major international guidelines, including those from EULAR, KDIGO and the ACR [114, 157, 158].

Although not yet approved for jSLE, clinical experience with this strategy is growing. Two recent real-world studies from China offered encouraging data. In a cohort of 47 patients, belimumab combined with standard therapy allowed for significantly lower glucocorticoid doses at 6 and 12 months and faster complement normalization, with similar remission and flare rates [166]. In a larger multicenter study (*N* = 101 participants), combination therapy with belimumab associated with higher complete renal response rates (94.1% versus 76.6%), greater rates of lupus low disease activity state (LLDAS) and DORIS remission, and more frequent glucocorticoid tapering (≤ 7.5 mg/day in 82.9% versus 30.4%), without serious adverse events [167].

### Treatments in Monogenic Lupus

#### JAK inhibitors

Because of the key involvement of type I IFN expression, several reports on the use of JAKi focus on early-onset monogenic forms of SLE and/or SLE-like disease, such as AGS [168–170]. Their efficacy in reducing systemic inflammation and preventing organ damage progression has led to recommendation for their use in AGS, chronic atypical neutrophilic dermatosis with lipodystrophy, elevated temperature/proteasome-associated autoinflammatory syndrome (CANDLE/PRAAS) and SAVI [171]. However, in AGS, JAKi have shown limited ability to halt neurological deterioration [172, 173]. The reasons for this are not fully understood, but may include the occurrence of irreversible and rapidly progressive neurological damage early in the disease course [174], as well as the limited ability of several molecules, including JAKi, to cross the blood-brain barrier [175].

Several case reports and small series have described the efficacy of JAKi in SAVI and other type I interferonopathies, although controlled studies are still lacking [176–178].

Tofacitinib has been used in two members of a family affected by chilblain lupus carrying a heterozygous mutation in the *TMEM173* gene (encoding STING) [179]. Similarly, cutaneous involvement improved markedly in response to tofacitinib in a family with an interferonopathy caused by compound heterozygous variants in *TREX1* [180]. Another report described a 13-year-old girl with jSLE and a novel heterozygous *TREX1* variant, successfully treated with baricitinib in combination with MMF [57]. A recent case of *BACH2*-related immunodeficiency and autoimmunity described a girl with early-onset jSLE, juvenile dermatomyositis and IgA deficiency, cutaneous and systemic symptoms improved with tofacitinib [18]. Collectively, these cases support the potential role of JAKi in selected paediatric lupus patients, particularly those with prominent cutaneous involvement and underlying strong interferon-driven pathophysiology.

#### Type I IFN-targeting treatment 

Evidence on the use of anifrolumab in monogenic lupus is limited yet promising. The first report on its use in a patient experiencing DNASE2 deficiency associated with reduced IFN scores and CRP levels, and a significant improvement of cytopenias [181]. In another study involving twelve patients with type I interferonopathies (SAVI = 3, CANDLE/PRAAS = 6, undifferentiated = 3), anifrolumab monotherapy in one case and in combination with baricitinib in eleven patients normalized the IFN scores, and improved systemic inflammation [182]. In a cohort of four patients with SAVI and one with CANDLE/PRAAS, anifrolumab normalized type I IFN-stimulated gene expression and improved clinical outcomes beyond what was achieved with JAKi alone. In two SAVI patients, anifrolumab monotherapy maintained type I IFN suppression, while in others, a combination with baricitinib showed additive effects, allowing tapering of glucocorticoids and JAKi [183]. Anifrolumab also demonstrated clinical efficacy in a SAVI patient who was refractory to JAK inhibitors and to the IFN-β-neutralizing monoclonal antibody dazukibart [184].

#### Reverse transcriptase inhibitors

Initial open-label data suggested that combined HIV-1 reverse transcriptase inhibitors (abacavir, lamivudine, and zidovudine) may reduce type I IFN signaling in AGS, supporting the hypothesis that endogenous retroelements contribute to disease pathogenesis [185]. However, findings from a recent multicenter crossover phase II trial did not confirm sustained effects; neither abacavir nor lamivudine alone reduced type I IFN scores at 6 weeks, while triple therapy induced a transient type I IFN score reduction at 3 weeks that was not maintained at 6 weeks, and did not associate with decreased IFN-α serum levels [185]. Moreover, no clinical benefit was demonstrated, and their therapeutic use remains uncertain. Additional RCTs are ongoing (NCT03304717, NCT05613868).

#### **Allogeneic hematopoietic stem cell transplantation (HSCT)**

 Allogeneic HSCT has been reported in a small number of patients with interferonopathies and related monogenic SLE-like diseases, with mixed results [186]. In an infant with PSMB9-associated CANDLE/PRAAS, HSCT resulted in complete remission [187], whereas a patient with PSMB4-associated disease required a second transplant to obtain full donor chimerism and restoration of proteasome function [188]. In POMP-related proteasome-related autoinflammation and immune dysregulation (PRAID), HSCT improved proteasome assembly, normalized T cell subset distribution, and resolved autoinflammation [189, 190]. In SAMD9L-associated autoinflammatory disease, three of four transplanted patients reached sustained remission, while one died from transplant-related pulmonary complications [191–193]. HSCT demonstrated curative potential in C1q-deficiency, with a 71% overall survival rate in a multicenter study [194], and achieved 86% survival in Activated Phosphoinositide 3-Kinase Delta Syndrome 1 (APDS1), improving immune dysregulation and lymphoproliferation [195]. Given the high procedure-related risk, HSCT may be considered in selected, treatment-refractory patients, particularly those with severe or progressive organ damage, where potential long-term benefits could outweigh the associated morbidity and mortality.

### Potential Future Treatments

Several emerging therapeutic strategies aim to directly target upstream drivers of type I IFN activation and innate immune dysregulation. Given the key role of TLR7/8 signaling in type I IFN-driven lupus pathogenesis, the phase II RCT from the WILLOW program (NCT05162586) evaluated the oral TLR7/8 inhibitor enpatoran. In patients with cutaneous lupus or SLE rash, enpatoran induced a dose-dependent improvement [196]. In moderate-to-severe SLE, it reduced type I IFN signatures and improved disease activity without added toxicity [197], supporting further exploration of TLR7/8 inhibition both in genetically complex and TLR7/UNC93B1-associated monogenic SLE.

Recent studies have reinforced the role of TRL7/MyD88-dependent signaling as a central driver of IFN activation and inflammation in SLE. In murine models, deletion or pharmacologic inhibition of MyD88 markedly reduced IFN-driven gene expression, immune activation, and end-organ damage [198]. Similarly, the selective MyD88 inhibitor ST2825 attenuated disease activity and proinflammatory cytokine production in lupus-prone mice [199]. These findings support MyD88 blockade as a promising therapeutic strategy to disrupt upstream innate immune pathways sustaining autoimmunity in SLE.

Defective degradation of neutrophil extracellular traps (NETs) due to loss-of-function variants in *DNASE1L3* or neutralizing antibodies promote type I IFN activation and systemic autoimmunity. In a recent study, recombinant DNASE1L3 and engineered DNASE1/DNASE1L3 variants resistant to antibody inhibition restored extracellular DNA degradation and reduced autoantibody production and tissue inflammation in experimental models [200]. These results provide a rationale for exploring DNASE1L3 replacement as a therapeutic approach in both monogenic DNASE1L3 deficiency and in polygenic SLE with secondary impairment of DNASE1L3 function.

Treatment with orthosteric STING inhibitors partially corrects the hyperactive conformation of SAVI-associated mutants, restoring key molecular interactions [201]. Recently, the irreversible STING antagonist LB244 demonstrated inhibition of STING-dependent signaling in vitro and in vivo [202], strengthening the rationale for STING blockade in disorders driven by aberrant STING-IFN axis activation including SAVI, AGS, COPA syndrome, and SLE [203–205]. Other components of this pathway, including cyclic GMP-AMP synthase (cGAS) and TANK-binding kinase 1 (TBK1), may represent additional therapeutic targets in these diseases [203].

### Treat-to-Target Approaches To Improve Outcomes in jSLE

Treat-to-Target (T2T) is a structured approach in which therapy is optimised until a pre-defined target, agreed with the patient/family, is achieved. This is then followed by ongoing monitoring and, when necessary, treatment adjustment. While T2T has been established in adult rheumatology, including rheumatoid arthritis [206], and part of SLE clinical trials [207, 208], efforts to develop a pediatric-specific framework started recently (2021). A multidisciplinary T2T taskforce, representing major pediatric rheumatology organisations (Pediatric Rheumatology European Society/PReS, Childhood Arthritis and Rheumatology Research Alliance/CARRA), nephrology, and adult rheumatology, developed consensus-based principles and points-to-consider for T2T in jSLE [209]. Moreover, pediatric-specific targets were defined, including Childhood Lupus Low Disease Activity State (cLLDAS) [210], cSLE Clinical Remission (cCR), and cSLE Clinical Remission Off-Steroids (cCR-0) [211]. Validation of targets confirmed their ability to accurately measure disease control in children, with attainment linked to fewer severe flares, lower damage risk, and comparable performance to adult targets [212].

Observational studies from cohorts across the world, including North America [213, 214], Thailand [215], China [216], and the Netherlands [217, 218] identified barriers to target attainment including the presence of LN [213–216], pre-existing damage [212, 213, 219], racial disparities [213], and lack of structured T2T implementation [220]. Facilitators include early MMF use [217], biologic therapy [216], and steroid weaning within three months of disease onset [217]. Importantly, target attainment associated with improved health-related quality of life (HRQOL) in the UK jSLE Cohort [221].

Recently, practical implementation of T2T approaches has begun, and a study involving 162 patients from the UK [222] demonstrated that T2T over a 12-month period associated with significant improvements in target attainment and reduced damage accrual at 12 months.

Integration of biomarkers alongside the T2T approach is also gaining interest, with two studies from the UK and Netherlands demonstrating associations between serum IFN-α levels and T2T states [223, 224].

While preliminary findings are encouraging, prospective multi-center studies are necessary to assess the feasibility and impact of T2T implementation in routine clinical practice, and to evaluate long-term outcomes. Such studies will be critical to establishing T2T as a standard of care in jSLE, and ensuring its scalability across diverse healthcare settings.

## Conclusions

Unprecedented advances in understanding the molecular pathophysiology of monogenic and genetically complex SLE has prompted the development of (preliminary) screening tools, molecular predictors or organ involvement and outcomes, and the development and (in some cases) licensing of new treatments. However, work remains to be done to sustainably improve the wellbeing of most patients with SLE. Key challenges are linked to ancestry-related differences in pathophysiology and clinical presentation that necessitates research involving all age groups (including children) and ancestries from all geographic regions.

## Data Availability

No datasets were generated or analysed during the current study.
